# Measuring the impact of a “Virtual Pediatric Trauma Center” (VPTC) model of care using telemedicine for acutely injured children versus the standard of care: study protocol for a prospective stepped-wedge trial

**DOI:** 10.1186/s13063-022-06996-1

**Published:** 2022-12-27

**Authors:** James P. Marcin, Daniel J. Tancredi, Joseph M. Galante, Tanya N. Rinderknecht, Brian M. Haus, Holly B. Leshikar, Marike Zwienenberg, Jennifer L. Rosenthal, Kendra L. Grether-Jones, Michelle Y. Hamline, Jeffrey S. Hoch, Nathan Kuppermann

**Affiliations:** 1 Department of Pediatrics, University of California, Davis, Sacramento, CA USA; 2 Department of Surgery, University of California, Davis, Sacramento, CA USA; 3 Department of Orthopedic Surgery, University of California, Davis, Sacramento, CA USA; 4 Department of Neurological Surgery, University of California, Davis, Sacramento, CA USA; 5 Department of Emergency Medicine, University of California, Davis, Sacramento, CA USA; 6 Department of Public Health Sciences, University of California, Davis, Sacramento, CA USA

**Keywords:** Pediatric, Trauma, Telemedicine, Telehealth, Family distress, Emergency department, Randomized controlled trial

## Abstract

**Background:**

The current standard of care in the treatment of children with physical trauma presenting to non-designated pediatric trauma centers is consultation with a pediatric trauma center by telephone. This includes contacting a pediatric trauma specialist and transferring any child with a potentially serious injury to a regionalized level I pediatric trauma center. This approach to care frequently results in medically unnecessary transfers and may place undue burdens on families. A newer model of care, the “Virtual Pediatric Trauma Center” (VPTC), uses telemedicine to make the expertise of a level I pediatric trauma center virtually available to any hospital. While the use of the VPTC model of care is increasing, there have been no studies comparing the VPTC to standard care of injured children at non-designated trauma centers with respect to patient- and family-centered outcomes. The goal of this study is to compare the current standard of care to the VPTC with respect to family-centered outcomes developed by parents and community advisory boards.

**Methods:**

We will use a stepped-wedge trial design to enroll children with physical trauma presenting to ten hospitals, including level II, level III, and non-designated trauma centers. The primary outcome measures are parent/family experience of care and distress 3 days following injury. Secondary aims include 30-day healthcare utilization, parent/family out-of-pocket costs at 3 days and 30 days after injury, transfer rates, and parent/family distress 30 days following injury. We expect at least 380 parents/families of children will be eligible for the study following an emergency department physician’s request for a level I pediatric trauma center consultation. We will evaluate parent/family experience of care and distress using previously validated instruments, healthcare utilization by family recollection and medical record abstraction, and out-of-pocket costs using standard economic analyses.

**Discussion:**

We expect that the findings from this study will inform other level I pediatric trauma centers and non-pediatric trauma centers on how to improve their systems of care for injured children. The results will help to optimize communication, confidence, and shared decision-making between parents/families and clinical staff from both the transferring and receiving hospitals.

**Trial registration:**

ClinicalTrials.gov Identifier: NCT04469036. Registered July 13, 2020 before start of inclusion.

## Administrative information

Note: the numbers in curly brackets in this protocol refer to SPIRIT checklist item numbers. The order of the items has been modified to group similar items (see http://www.equator-network.org/reporting-guidelines/spirit-2013-statement-defining-standard-protocol-items-for-clinical-trials/).

**Table:** 

Title {1}	Measuring the Impact of a “Virtual Pediatric Trauma Center” (VPTC) Model of Care Using Telemedicine for Acutely Injured Children Versus the Standard of Care: Study Protocol for a Prospective Stepped-Wedge Trial
Trial registration {2a and 2b}.	ClinicalTrials.gov Identifier: NCT04469036. Registered: July 13, 2020 https://trialsearch.who.int/Trial2.aspx?TrialID=NCT04469036
Protocol version {3}	Version 2; 5-21-2021.
Funding {4}	This research is funded by the Patient-Centered Outcomes Research Institute (PCORI) IHS-2019C1-16093.
Author details {5a}	James P Marcin, MD, MPH. Department of Pediatrics, University of California, Davis, Sacramento, California. Daniel J Tancredi, PhD. Department of Pediatrics, University of California, Davis, Sacramento, California. Joseph M Galante, MD. Department of Surgery, University of California, Davis, Sacramento, California. Tanya N Rinderknecht, MD. Department of Surgery, University of California, Davis, Sacramento, California. Brian M Haus, MD. Department of Orthopedic Surgery, University of California, Davis, Sacramento, California. Holly B Leshikar, MD, MPH. Department of Orthopedic Surgery, University of California, Davis, Sacramento, California. Marike Zwienenberg, MD. Department of Neurological Surgery, University of California, Davis, Sacramento, California. Jennifer L Rosenthal, MD, MAS. Department of Pediatrics, University of California, Davis, Sacramento, California. Kendra L Grether-Jones. Department of Emergency Medicine, University of California, Davis, Sacramento, California. Michelle Y Hamline, MD, PhD, MAS. Department of Pediatrics, University of California, Davis, Sacramento, California. Jeffrey S Hoch, PhD. Department of Public Health Sciences, University of California, Davis, Sacramento, California. Nathan Kuppermann, MD, MPH. Department of Emergency Medicine and Department of Pediatrics, University of California, Davis, Sacramento, California.
Name and contact information for the trial sponsor {5b}	Patient-Centered Outcomes Research Institute (PCORI) 1828 L Street NW, Suite 900 Washington, CD 20036 Phone: (202) 827-7700 Email: info@pcori.org
Role of sponsor {5c}	The study funder (PCORI) has no role in the design of the study and collection, analysis, and interpretation of data and in writing the manuscript.

## Introduction

### Background and rationale {6a}

The American College of Surgeons Committee on Trauma (ACS-COT) has been committed to improving the care provided to injured patients since 1922. An essential component of their efforts has been the creation of standards for trauma facilities and a tiered trauma care system. As detailed in the ACS-COT published guidelines, *Resources for Optimal Care of the Injured Patient* [1], these standards outline the five levels of trauma facilities that define varying levels of commitment, readiness, resources, policies, patient care, and performance improvement [1]. A level I trauma center is the highest designation and is only granted to hospitals that can provide the highest level of care to all injured patients. The ACS-COT Trauma Center Verification process has been instrumental in improving outcomes among injured children and adults and has become the national model of trauma care coordination as well as the prototype for trauma care internationally [2, 3].

While the regionalization of trauma care has resulted in improved outcomes [3–5], the current standard of care has created disparities in access for patients injured in geographically isolated locations. When children living in non-metropolitan communities are seriously injured and present to non-pediatric trauma center emergency departments (EDs), they are transferred to the regionalized level I pediatric trauma center. In more than half of the states in the USA, most children live more than 30 miles from a designated level I pediatric trauma center. Currently, there are more than 41 million children in the USA that have poor access to trauma care, living more than 30 miles from a pediatric trauma center. It is these children who would most benefit from a system of care that mitigates the differences in access to high-quality care for injured children [6–8].

Because the current regionalization of trauma centers has created differences in access, many pediatric trauma experts, including health policy makers, health services researchers, and front-line clinicians have advocated for the use of telemedicine. This technology allows level I pediatric trauma center expertise to be transmitted to receiving EDs where most children with physical trauma initially present [9–13]. This newer system of care has been referred to as the “Virtual Pediatric Trauma Center” (VPTC) and is increasingly used by hospitals and EDs throughout the country [12, 14]. The VPTC creates a model of care that connects EDs in non-level I trauma centers using telemedicine to bring expert pediatric trauma care to the bedside of injured children, regardless of where the patient presents. While this newer model of care enables participation of parents/families in the initial phases of trauma care, there is conflicting and limited data comparing this model to the current standard of care as it relates to parent/family-experience and distress, healthcare utilization, and financial impact on parents/families [15, 16].

As evidence, in preparation for this study, we conducted three meetings with community advisory boards which laid the foundation for the study design and evaluation. We met with members from each of these boards to focus on parent/family-centered measures. Our team of clinical investigators, consortium hospital partners, and two broadly representative community advisory boards believed that comparing these two models of care would provide important evidence on the best approach to address problems facing families needing specialized trauma care for their children. Having the core members of a regionalized level I pediatric trauma center available virtually at the bedside of injured children via telemedicine has the potential to enhance parent and family involvement in care, participate in shared decision-making, and may reduce parent/family distress and unnecessary and financially burdensome hospital transfers. Alternatively, parents and families may prefer to err on the side of safety and have their injured children immediately transferred to the regional level I pediatric trauma center. In this case, delaying or avoiding the transfer of an injured child to a better equipped and staffed facility could result in increased parent/family distress, healthcare utilization, and out-of-pocket costs. Therefore, a rigorous comparison of the two prevailing models of care is needed to inform the best model of trauma care in children.

### Objectives {7}

The focus of this comparative effectiveness study is to evaluate the current standard of pediatric trauma care and the VPTC model of care with regards to the parent/family experience, parent/family distress, healthcare utilization, and out-of-pocket cost burden. To better understand how these two models of care impact these parent/family-centered outcomes, we are conducting a comprehensive study on these outcomes among a diverse cohort of 10 ED located within rural and underserved non-pediatric trauma centers.

Our primary outcome measures are parent/family experience of care and distress 3 days following injury requiring an ED visit in a non-pediatric trauma center under the current standard model of care versus the VPTC model of care. Our null hypothesis is that measures of parent/family experience of care and measures of distress will be similar between the two models of care. Our secondary outcome measures are as follows: (1) 30-day healthcare utilization (our null hypothesis is that hospitalizations, re-hospitalizations, primary and specialty care visits will be similar between the two models of care); (2) out-of-pocket costs and financial burdens experienced by parents/families at 3 days and 30 days following a childhood injury requiring an ED visit in a non-pediatric trauma center (our null hypothesis is that out-of-pocket costs and financial burdens for parent/families will be similar between the two models of care); (3) frequency of transfers from a non-pediatric trauma center (our null hypothesis is that the frequency of transfers will be similar between the two models of care); and (4) parent/family distress 30 days following a childhood injury (our null hypothesis is that measures of parent/family distress will be similar between the two models of care).

### Trial design {8}

This is a prospective, stepped-wedge trial with superiority framework.

## Methods: participants, interventions, and outcomes

### Study setting {9}

Participating hospitals include one level I pediatric trauma center within a quaternary university hospital (UC Davis Health) and 10 non-children’s hospitals that are either an adult-designated Level II trauma center (*N* = 2), an adult-designated Level III trauma center (*N* = 2), or a non-designated trauma center hospital (*N* = 6). All participating hospitals are located in Northern California.

### Eligibility criteria {10}

All children (< 18 years old) presenting to participating EDs with acute injuries during the study period that receive a consultation from a UC Davis Health trauma surgeon, orthopedic surgeon, or neurosurgeon are eligible for inclusion. Exclusion criteria include children who are wards of the state or do not have a parent present at the participating hospital, children who receive cardiopulmonary resuscitation prior to presenting to any of the participating hospitals, and children who die within 3 days following the acute injury. The consultations that are part of the standard model of care (telephone) and the VPTC (telemedicine) model of care are conducted by one of the specialist physicians (trauma surgeon, orthopedic surgeon, neurosurgeon) or by a trauma nurse practitioner.

### Who will take informed consent? {26a}

Consent for participation (survey completion) will be obtained by trained, research team coordinators. The parents/caregivers of the injured child will sign the informed consent if they agree to participate in the survey completion process.

### Additional consent provisions for collection and use of participant data and biological specimens {26b}

N/A. This is a comparative effectiveness trial evaluating a systems-level intervention; the system-level interventions qualify as current standards of care.

### Interventions

#### Explanation for the choice of comparators {6b}

This is a comparative effectiveness trial—both the “standard of care” and the “virtual pediatric trauma center” models of care are currently practiced and are acceptable approaches within the current standard of care for pediatric trauma patients.

#### Intervention description {11a}

Both the current standard of care (telephone consultation) and the VPTC model of care (telemedicine consultation) are provided by UC Davis Health trauma surgeons, pediatric orthopedic surgeons, or pediatric neurosurgeons. Consultations are initiated after a referring ED physician at a participating ED calls the UC Davis Health transfer center and requests a consultation for an injured child. For the current standard model of care, the consultation is completed over the telephone. For the VPTC model of care, following the brief telephone conversation, a telemedicine consultation is conducted. Telemedicine consultations use synchronous audio-video communication and involve the patient’s parents/families, and when available, the referring physician, bedside nurse, and/or respiratory therapist. Telemedicine units in the EDs consisted of a pole-mounted, high-resolution videoconferencing unit with pan-tilt-zoom capabilities that use the internet for high-definition video (minimum 1Mbps, 720p). Telemedicine capabilities at UC Davis Health are accessible via workstation computers using videoconferencing software, a headset, and a webcam.

#### Criteria for discontinuing or modifying allocated interventions {11b}

In all cases, a telephone consultation is conducted between physicians at the participating ED and physicians/providers at UC Davis Health. Whether the encounter occurs during a period where the standard model of care or VPTC model of care is supposed to be allocated, physicians and providers from either the participating ED or UC Davis Health could use the model of care of their choice. Specifically, providers could decline to use the VPTC model of care (telemedicine) at their discretion. In addition, the parents/families could decline to participate in the VPTC model of care at their discretion.

#### Strategies to improve adherence to interventions {11c}

The continuous monitoring and success of this study is the primary responsibility of the principal investigator and multidisciplinary team of co-investigators with expertise in pediatric trauma, telemedicine, community engagement, implementation science, and health services research. The study team will conduct bi-weekly meetings to review every individual study patient encounter. With every eligible study patient encounter, a research analyst will reach out to the relevant parties involved via email or telephone to discuss any barriers to intervention implementation. The feedback is shared with the study team, challenges are discussed, and strategies used to improve intervention adherence. Strategies to improve adherence include automated reminders to UC Davis Health transfer center personnel regarding participating EDs, transfer center intake templates with the names of the participating EDs, and text and email reminders to the teams corresponding with participating EDs. All interventions are tracked and monitored. Monthly recruitment, survey completion, and intervention adherence charts are updated and shared with the study team during meetings to monitor adherence.

#### Relevant concomitant care permitted or prohibited during the trial {11d}

N/A. There is no relevant concomitant care prohibited during the trial.

#### Provisions for post-trial care {30}

N/A. As a system-level comparative effectiveness trial, both accepted models of care (telephone and the telemedicine models of care) will be administered as usual. Post-trial care is not required for this study protocol.

#### Outcomes {12}

Our primary outcome measures are the parent/family experience of care and distress 3 days following a childhood injury. Our secondary outcome measures are (1) 30-day healthcare utilization, (2) the out-of-pocket costs and financial burdens experienced by parents/families at 3 days and 30 days following a childhood injury, (3) the frequency of transfers in a non-pediatric trauma center, and (4) parent/family distress 30 days following a childhood injury.

To measure parent/family experience of care, we will use questions from the CAHPS Child Hospital survey [17] with additional questions developed with our community advisory groups and our parent stakeholder co-investigator (Table 1). This measure was selected as patient and parent/family experience of care has increasingly been used as a clinical outcome measures in emergency medicine research [18–20] and correlates with many objective clinical outcomes [21].

**Table 1:** Questions from the Consumer Assessment of Healthcare Providers and Systems Survey

Questions related to ED experience with providers	Questions related to ED discharge
During the first day of this emergency department stay, were you asked to list or review all of the prescription medicines your child was taking at home?	Before your child left the emergency department, did a provider ask you if you had any concerns about whether your child was ready to leave?
During the first day of this emergency department stay, were you asked to list or review all of the vitamins, herbal medicines, and over-the-counter medicines your child was taking at home?	Before your child left the emergency department, did a provider talk with you as much as you wanted about how to care for your child’s health after leaving the emergency department?
During this emergency department stay, how often did your child’s nurses listen carefully to you? During this emergency department stay, how often did your child’s nurses explain things to you in a way that was easy to understand?	Before your child left the emergency department, did a provider or emergency department pharmacist explain in a way that was easy to understand how your child should take these new medicines after leaving the emergency department?
During this emergency department stay, how often did your child’s nurses treat you with courtesy and respect?	Before your child left the emergency department, did a provider or emergency department pharmacist explain in a way that was easy to understand about possible side effects of these new medicines?
During this emergency department stay, how often did your child’s doctors listen carefully to you? During this emergency department stay, how often did your child’s doctors explain things to you in a way that was easy to understand?	A child’s regular activities can include things like eating, bathing, going to school, or playing sports. Before your child left the emergency department, did a provider explain in a way that was easy to understand when your child could return to his or her regular activities?
During this emergency department stay, how often did your child’s doctors treat you with courtesy and respect?	Before your child left the emergency department, did you get information in writing about what symptoms or health problems to look out for after your child left the emergency department?
During this emergency department stay, how often were you given as much privacy as you wanted when discussing your child’s care with providers?	When deciding whether your child should be transferred, admitted, or discharged home, did you feel like you were a part of the decision-making process?
During this emergency department stay, how often did providers keep you informed about what was being done for your child?	

Responses: “Yes, definitely” “Yes, somewhat” “No”

To measure parent/family distress, we will use a subset of the state anxiety portion of the State-Trait Anxiety Inventory Form Y [22]. The surveys used to measure experience of care and parent/family distress will both be administered to eligible parents/families 3 days following the injury to measure the primary outcomes. We will also plan to collect a subset of the State-Trait Anxiety Inventory Form Y from families at 30 days as a secondary outcome measure to determine longer-term distress and anxiety (Table 2).

**Table 2:** Questions from the State-Trait Anxiety Inventory Form Y

I feel calm	I feel at ease	I feel frightened	I am jittery	I am worried
I feel secure	I feel upset	I feel comfortable	I feel indecisive	I feel confused
I am tense	I am presently worrying over possible misfortunes	I feel self-confident	I am relaxed	I feel steady
I feel strained	I feel satisfied	I feel nervous	I feel content	I feel pleasant

Responses: “Not at all” “Somewhat” “Moderately so” “Very much so”

The measure of 30-day healthcare utilization includes both hospital and ambulatory clinic encounter data. These data will be obtained by both medical record review and parent/family surveys. Hospital and clinic encounter healthcare utilization will include hospital length-of-stay and the Universal Billing form-04 (summary billing statements). Parent/family survey data on healthcare utilization will be collected at the time of the 3-day and 30-day follow-up.

To measure out-of-pocket costs experienced by parents/families 3 days and 30 days post injury, surveys will include a modified version of the iMTA Productivity Cost Questionnaire (iPCQ). The survey (Table 3) includes three modules measuring productivity losses of paid work due to (1) absenteeism and (2) presenteeism and productivity losses related to (3) unpaid work [23]. To measure the frequency of transfers, we will identify the patient disposition following the encounter at the participating ED. Patient disposition will include those patients transferred to UC Davis Health, those patients admitted to the local hospital from the participating ED, and those patients transferred to another hospital, as well as those patients discharged home from the participating ED. To further define transfers, we will apply a validated method to identify transfers that are potentially avoidable [24, 25]. Potentially avoidable transfers will be defined as patients transferred and discharged within 24 h without receiving a specialized diagnosis or procedure.

**Table 3:** Questions obtained from the iMTA iPCQ

Question	Selectable Response (if applicable)
What do you do?	• Go to school • Employed • Self employed • housewife/househusband, unemployed, unable to work for • Am retired or on a pre-pension plan • I do something else:
How many hours a week do you work?	
During the last 4 weeks have there been days in which you worked but during this time were bothered by physical or psychological problems?	• Yes • No
Were there days in which you were forced to do less unpaid work because of physical or psychological problems? Only days in the last four weeks.	
Do you have a paying job?	
How many days a week do you work?	
How many days at work were you bothered by physical or psychological problems?	
How many days did this happen in the past four weeks?	
What is your occupation?	
Have you missed work in the last 4 weeks as a result of your CHILD being sick?	• Yes • No • How many days of work have you missed in the past 4 weeks due to your child being sick
On the days that you were bothered by these problems, was it perhaps difficult to get as much work finished as you normally do? On these days how much work could you do on average?	
Imagine that somebody, for example your partner, family member or friend helped you on these days, and he or she did all the unpaid work that you were unable to do for you. How many hours on average did that person spend doing this on these days?	

Questions slightly modified from iMTA iPCQ to be relevant to questions for parents/families following ED encounter

#### Participant timeline {13}

Participant timeline, including time of enrollment and outcome assessments, is provided in Table 4.

**Table 4:** Schedule of enrolment, intervention, and assessments

	Study period						
	Enrolment	Allocation	Post-allocation				
Timepoint	Baseline	0	Day 3	Day 30	Day 60	Day 90	24 months
**Enrolment:**							
**Eligibility screen**	X						
**Medical record review**		X					
**Demographics obtained from EMR**		X					
**Interventions:**							
**Standard of care versus VPTC per stepped-wedge design**		X					
**Assessments:**							
**CAHPS Child Hospital survey**			X				
**Modified iMTA Productivity Cost Questionnaire**			X	X			
**State-Trait Anxiety Inventory Form Y**			X	X	X	X	
**End of Study**							X

*Abbreviations*: *EMR* Electronic medical record, *VPTC* Virtual pediatric trauma center, *CAHPS* Consumer Assessment of Healthcare Providers and Systems, *iMTA* Institute for Medical Technology Assessment

#### Sample size {14}

To calculate the required sample size accounting for the stepped-wedge cluster-randomized design and multilevel data analytics strategy, we performed 2-tailed (alpha = 5%) power analyses using simulated data in SAS. We varied within-ED intracluster correlation from 5 to 10% (to ensure power across a plausible range of cluster effects). This was done assuming that a cubic polynomial would be needed to control for calendar time effects, a highly conservative assumption because the cubic polynomial accounts for more variation (i.e., is collinear with) in the stepped-wedge treatment indicator than would a simple linear term for calendar time [26–31]. In our previous research, we found the mean overall parent/family experience of care on a previously validated seven-point Likert item was 5.60 (95% CI, 5.42–5.79) among 46 patients receiving tele-emergency consultations and 5.20 (95% CI, 5.07–5.34) among 28 patients receiving telephone consultations and an estimated standardized effect size of 0.77 [32]. We specified that a minimum meaningful difference for the continuous survey outcomes, including experience of care and the state-trait anxiety index subscale, would be a standardized effect size of two thirds (0.67).

Given the proposed sudy duration and previous data on pediatric trauma consultations, we expect approximately 380 pediatric trauma consultations from the 10 participating sites over the 2-year enrollment period. Using these data for our primary outcome measures, we determined that we would have at least 83.4% power to detect this anticipated effect on experience of care and parent/family distress, even if the participation rate were only 50% of eligible participants. However, we anticipate a retention rate of 90% at 30 days, 70% at 60 days, and 60% at 90 days, ensuring ample power to detect the stated effect size of 0.67 standard deviation.

Regarding secondary outcomes, our power analyses for healthcare utilization and out-of-pocket costs demonstrated greater than 80% power to detect standardized effects of 0.67 SD, even with data on only 50% of eligible participants. Regarding the statistical power to detect differences in transfer rates, we again accounted for the stepped-wedge design, the hierarchical nesting of patients within sites, and the regression strategy. We implemented Hussey and Hughes power and sample size procedures via simulated data in SAS, assuming a cubic polynomial to control for period effects for each of the following power calculations and a within-site intracluster correlation coefficient of 5% [26–28]. Currently, almost all patients fulfilling study eligibility criteria are transferred, so we sought to detect a reduction of 20 percentage points in transfers. We determined that we would have greater than 84% statistical power to detect this reduction, even with an intracluster correlation coefficient as high as 10%.

#### Recruitment {15}

Our team will identify all eligible patients using transfer center data collected on all calls to UC Davis Health for consultations related to pediatric trauma and injury. Data are easily identifiable using the electronic health record on every eligible patient from our participating sites.

Several methods of patient recruitment will be used, primarily based on the location of the patient, to ensure that all eligible patients are approached for participation. ED clinical research coordinators will approach parents/families if the child is transferred to the UC Davis Health ED. For families who are admitted directly to the hospital, research coordinators will primarily use remote recruiting methods (telephone and text messaging) to approach families within 2 days of the injury. In-person recruitment by research coordinators for patients who have been admitted has been variable due to COVID-19 precautions. For patients who have a consultation following an acute injury but are not transferred, research coordinators use remote recruitment methods via telephone and text messaging.

### Assignment of interventions: allocation

#### Sequence generation {16a}

To compare our parent, family, community, and provider informed outcomes between the current standard of care and the VPTC approaches, we will use a prospective stepped-wedge trial design [28]. This design has important advantages over alternative designs. First, with the stepped-wedge design, changes in the quality and standards of pediatric trauma care over time can be accounted for, unlike a simple pre-test, post-test study design where changes in care could be confounded by temporal changes and secular trends. Second, in common with a cluster randomized design, and in contrast to patient-randomized designs, our design minimizes contamination bias that could arise among researchers and participants when patients at the same site have been concurrently randomized to two different models of care. In addition, the stepped-wedge design has an important statistical power advantage over a parallel-groups cluster-randomized trial. The latter design suffers a loss of statistical power that arises from within-cluster correlation, even when this correlation is as modest as 5%, a typical value for process-of-care outcomes [33]. The presence of positive intra-cluster correlation has the opposite effect in a stepped-wedge design, because in that design, the contrast of interest is a “within-cluster” contrast, so that the cluster serves as its own control, increasing the effective sample size and resulting statistical power [34].

There are 66 hospital EDs that transfer children with trauma to the UC Davis Children’s Hospital Level I pediatric trauma center. A stratified sample of 10 EDs was selected to include two hospitals designated as adult level II trauma centers, two hospitals designated as adult level III trauma centers, and six hospitals without a trauma designation. Five sites were selected because they are in rural communities, as designated by the State of California’s Office of Statewide Health Planning and Development. The site selection process also included consideration of the patient population served to maximize diversity in racial/ethnic representation and socioeconomic status.

To promote balance, we formed two blocks of five hospitals, with each block containing one randomly assigned level II trauma center, at least one randomly assigned Level III trauma center, and at least two randomly assigned non-designated centers. After a 6-month pre-implementation period, the study was initiated with all hospitals beginning in the standard of care condition (receiving telephone consultation) and with patients enrolled for 13 consecutive 8-week periods. The five hospitals in the first block were randomly arranged and enumerated from 1 to 5 and, similarly, the five in the second block were randomly enumerated 6 to 10. At the end of each of the 8-week periods, one hospital ED will switch to the VPTC model of care condition (telemedicine consultation), according to its randomly assigned enumeration.

#### Concealment mechanism {16b}

N/A. Participating sites will know the date they switch from the standard-of-care condition to the VPTC model-of-care condition to use the appropriate model of care.

#### Implementation {16c}

**Fig. 1:**
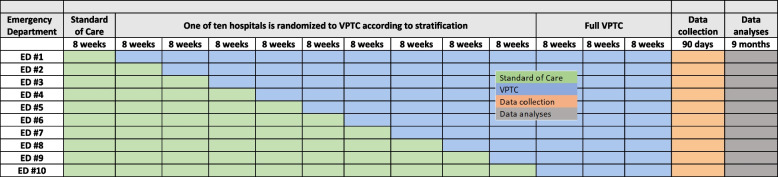
Stepped wedge enrollment and timeline

As noted in “Sequence generation {16a}” section, we will use a stepped wedge trial design. After a 6-month pre-implementation period, the study will begin with all hospitals beginning in the standard of care condition and with patients enrolled for 13 consecutive 8-week periods. At the end of each of the 8-week periods, one hospital ED will switch to the VPTC model of care condition, according to its randomly assigned enumeration previously described. Following the 2-year (13 × 8 weeks) enrollment period, there will be a 6-month post-implementation period for data analysis and dissemination. See Fig. 1.

### Assignment of interventions: blinding

#### Who will be blinded {17a}

This study involves two acceptable and existing models of care. The date and ED location where a child presents will determine which model of care the child receives. The patient/family will receive either the standard of care or VPTC model of care. Trial participants and care providers will not be blinded. Data analysts and statisticians will all be blinded once the survey and electronic health record data has been entered into the database. This will be done by labeling the care models without reference to the standard of care (telephone) or the VPTC (telemedicine).

#### Procedure for unblinding if needed {17b}

N/A. We have implemented a stepped-wedge trial design in which the patient/family and care providers are not blinded to the model of care being provided.

### Data collection and management

#### Plans for assessment and collection of outcomes {18a}

All parents/family members of eligible pediatric trauma patients will be eligible to participate in the survey portion of the study. The UC Davis Health research team will attempt to contact parents/family members within 3 days following the injury to be able to administer the 3-day and 30-day surveys. The research team will review the informed consent document with the parent/family member which explains the purpose of the surveys—to determine the parent/family experience with pediatric trauma care, the parent/family level of distress, the patient utilization of healthcare, and out-of-pocket costs following injury.

The questions from the CAHPS Child Hospital survey [17] that will be used to measure parent/family experience of care are well established and validated measures [35] and has been shown to correlate with many objective clinical outcomes [21]. Similarly, the instrument that we will use to measure parent/family distress, the State-Trait Anxiety Inventory Form Y, has been shown previously to be a reliable and valid measure of anxiety and distress [22, 36]. The hospital and ambulatory clinic encounter data that will be used to measure 30-day healthcare utilization will be collected by medical record review and will be compared to and validated with parent/family 3-day and 30-day surveys. Our measure out-of-pocket costs experienced by parents/families 3 days and 30 days post injury will be determined by the iMTA Productivity Cost Questionnaire (iPCQ). This survey has also been shown to understandable and valid [23, 37]. Finally, to measure the frequency of transfers and potentially avoidable transfers, we will use the disposition and transfer data from the participating ED and a previously validated method and measure of potentially avoidable pediatric interfacility transfers, respectively [24, 25].

#### Plans to promote participant retention and complete follow-up {18b}

Participation in this trial is voluntary and refusal to participate will involve no penalty or change in care, and the privacy of participants will be maintained as the surveys do not contain identification of participants. Following consent, research coordinators will contact parents/families via telephone and/or text to complete surveys. If the parent/family wishes to conduct the surveys over the telephone, this will also be supported. This was a strategy that was recommended by our community advisory boards. Additionally, as suggested by our community advisory boards and parent co-investigator, parents/family members will have the option to complete the survey over text message or e-mail. Families will be contacted as soon as possible within 72 h of the initial injury in order to complete the 3-day surveys. Outreach to the parent/family will continue for up to 1 week during each survey period (3-day, 30-day, 60-day, and 90-day surveys) via a combination of email, text messages, or phone calls. Outreach is discontinued at any point if a family declines to participate.

#### Data management {19}

All children (< 18 years old) presenting to participating hospital EDs during the study period receiving a pediatric trauma consultation from a relevant UC Davis clinician will be included in the study. Eligible pediatric trauma patients will be retrospectively identified by the research team using the existing UC Davis Health transfer center children’s request log in EPIC which records all consultations (telephone and telemedicine) with referring hospitals. A study identification number will be assigned to each patient such that no patient identifiers will be included in the database. Then, using the study identification number, parent/family survey data will be entered into a secure REDCap database by trained research personnel.

#### Confidentiality {27}

This study was deemed by the UC Davis Health human subjects review committee to only involve minimal risk related to the potential loss of confidentiality. This research protocol involves care that is considered standard. Data will be stored such that there will be no link to the individual’s identity and medical records. Survey and medical chart data will not include any information that identifies the participants when the investigator team accesses it. Additionally, once the link between the study identification number and the patient’s records is destroyed, there will be no way to identify any study participant. All data will be stored on secure, password-protected, encrypted UC Davis Health computers in REDCap.

#### Plans for collection, laboratory evaluation, and storage of biological specimens for genetic or molecular analysis in this trial/future use {33}

N/A. Our trial does not require collection of biological specimens or laboratory evaluation.

### Statistical methods

#### Statistical methods for primary and secondary outcomes {20a}

Our team includes a senior faculty biostatistician and a senior faculty health economist, as well as clinical epidemiologists. Our primary outcomes—parent/family self reported experience of care and distress—will be analyzed with regression analyses using a generalized linear mixed model framework for multilevel data (patients nested within referring hospitals). We will use random effects for hospitals specified to account for unmeasured sources of between-unit heterogeneity [38–40] and robust variance estimators. A within-site time-varying binary indicator (standard-of-care versus VPTC) will be included in the model to permit estimating the key contrasts of interest. For our primary outcomes (parent/family experience of care and parent/family distress), we will use linear-normal mixed models effect sizes to determine adjusted mean differences. We will use similar methodolgy for other continuous outcome variables including out-of-pocket costs at 3 and 30 days from injury. For the secondary outcome measures of healthcare utilization and transfer, we will use count and binary outcomes fitted with Poisson and logistic regression models, respectively. Effect sizes will be rate ratios and adjusted odds, respectively.

For outcomes assessed both 3 days and 30 days after injury, we anticipate estimating time point-specific contrasts of the standard-of-care and VPTC models of care. To do this, models will also include a binary indicator for time point (3 day vs. 30 day) as well as interactions of time point with the time-varying treatment group indicator. Additional independent variables will include a parsimonious and pre-specified set of patient-level effects to account for study design issues and to improve the precision of estimated effects, accounting for known sources of heterogeneity [39, 41, 42]. To account for confounding by calendar time in our stepped-wedge design, [28] we will compute each patient’s date at enrollment in the study and use fractional polynomial modeling to determine the order of polynomial (linear, quadratic, cubic, etc.) to specify the calendar date of enrollment effects, to account for temporal effects [43]. A senior health economist will conduct the economic analyses relevant to the financial burdens experienced by parent/family members.

#### Interim analyses {21b}

N/A. There are no planed interim analyses.

#### Methods for additional analyses (e.g., subgroup analyses) {20b}

Subgroup analyses: We plan to include the following empirically driven inquiries in our subgroup analyses: (1) injury severity; (2) injury type; (3) distance from hospital; and could also include (4) patient characteristics (e.g., race/ethnicity, age, insurance type, etc.). The first three would be confirmatory in nature and the latter exploratory. Rationale: For injury severity: lower-severity children with trauma may experience less with respect to primary and secondary outcomes, including parent/family distress and out-of-pocket costs. For injury type: Variation is expected with injury type, with some injury types (traumatic brain injury, isolated orthopedic injuries) creating more/less parent/family distress. We will categorize injury types into Clinical Classifications Software diagnostic groupings based on principal diagnoses. Distance from hospital: distance is expected to correlate with higher out-of-pocket costs. Individual patient characteristics: race, ethnicity, age, and insurance type will also be examined. Social determinants of health will also be included in exploratory analyses. We anticipate that additional subgroup analyses may be developed in partnership with our parent co-investigator, family advisory board, and community advisory committee, the three pillars of our Community and Stakeholder Engagement Plan. To maintain rigor, subgroup analyses will include formal assessment of whether treatment effects are heterogeneous among the levels of each variable used to define subgroups, by including and assessing relevant interaction terms involving the subgroup variable and the time-varying VPTC indicator.

#### Methods in analysis to handle protocol non-adherence and any statistical methods to handle missing data {20c}

We will measure, analyze, and report the intervention assignment adherence. Outcomes will be measured using three methods: (1) intention-to-treat analyses, (2) treatment received analyses, and (3) per-protocol analyses. Fidelity and assignment adherence will be assessed by reviewing the UC Davis Health transfer center database, which includes documentation on all communication events. Intervention assignment adherence will be assessed by tracking the proportion of eligible encounters for whom the protocol assignment (telemedicine vs. telephone) is followed.

Based on experience, we anticipated a relatively small amount of missing data, which would arise from failure to complete follow-up surveys or from intermittently missing clinical or survey items. We will characterize nonresponse and missing data patterns between the two arms of the study. We will follow a principled approach to addressing missing data that includes conducting primary analyses using the generalized linear modeling framework under a missing-at-random assumption and supplementing the primary analysis with sensitivity analyses that make alternative assumptions about the missing data [44]. In trial reports, we will provide a range of intervention estimates, graded according to the plausibility of the missing data assumption. We anticipate that the most plausible assumption will be missing at random, and we will use a multiple imputation framework within our mixed-effects modeling strategy to estimate intervention effects under this assumption [45]. Alternative missing data assumptions will also be estimated within a multiple imputation framework, by varying the imputation model accordingly.

#### Plans to give access to the full protocol, participant level-data, and statistical code {31c}

Investigators on this project have professional relationships and/or leadership roles with many national organizations with whom we will partner to disseminate findings and assist in widespread implementation. The two models of care being compared in this study are both acknowledged by the ACS-COT which will help advocate for change based on our findings (https://www.facs.org/quality-programs/trauma). PECARN is a national pediatric emergency care research network supporting multi-center studies and meaningful informational exchange (http://www.pecarn.org). Our team will leverage existing relationships with PECARN and trauma societies to distribute results of our findings. With regard to telemedicine, there is a consortium of Telehealth Resource Centers representing every state and territory in the country (https://www.telehealthresourcecenter.org/) to assist in all aspects of operations, reimbursement, training, and program development. Last, we will discuss results with the National Telehealth Policy Resource Center, whose mission is to provide resources to all states and territories regarding the legal and regulatory issues surrounding the use of telemedicine (http://www.cchpca.org/). In addition to disseminating results through national partnerships, the investigators are committed to publishing manuscripts and presenting results at national meetings and conferences.

### Oversight and monitoring

#### Composition of the coordinating center and trial steering committee {5d}

This project has brought together a diverse team of experienced researchers, expert clinicians, an insightful parent co-investigator, and two community advisory boards to serve as the primary research team for the study.

##### Patient-centered team

The parent co-investigator’s perspective as a parent of a child who experienced trauma will ensure that our project is patient- and family-centered and will allow us to implement the project, interpret our results and disseminate our findings in a way that is meaningful to patients, parents, and caregivers. The Center for Healthcare Policy and Research (CHPR) at UC Davis will help us organize a Family Advisory Board, consisting of parents from the communities served by the 10 partner hospitals. This group will provide valuable oversight of our study and will provide the perspective of parents from the rural and urban areas to ensure that our study is responsive to the unique viewpoints of these regions. The Pediatric Emergency Care Applied Research Network (PECARN) Pediatric Research in Injuries and Medical Emergencies (PRIME) research node’s Community Advisory Committee will provide community and provider stakeholder input, which will ensure that our study can be replicated in a variety of settings, and that our findings are relevant to the larger medical community.

##### Participating EDs

The hospital EDs chosen to participate in the study were selected to include a variety of rural and underserved populations from wide geographic areas and to represent a range of designated adult Level II and III trauma centers, as well as those without a trauma designation. We have existing telemedicine relationships with each participating hospital. Each site will have extensive oversight from the PI and research analyst team, who will work to maintain relationships, provide education (along with the parent co-investigator and other study team members), and ensure successful data collection with the help of the research data analyst. The PI and research analyst team will work with the sites and the study team to ensure that all study milestones are met on time. The research team and its site partners will also anticipate problems through regular research meetings with each site. A research analyst on the study will provide data monthly to the partner hospitals, as well as reach out in real-time to troubleshoot any issues.

#### Composition of the data monitoring committee, its role and reporting structure {21a}

N/A. Because this study was determined to be minimal risk, we will not include a data safety and monitoring board. To ensure that no adverse events take place, we will implement a monitoring plan to review several sources of data of study participants. This will ensure we identify if any are subsequently treated in any consortium EDs, readmitted to consortium hospitals, or transferred to UC Davis Health for care related to the index injury.

#### Adverse event reporting and harms {22}

N/A. While there is the potential risk of loss of confidentiality, for all data, only study personnel will have access to these materials. All data will be destroyed 7 years after completion of the study.

#### Frequency and plans for auditing trial conduct {23}

N/A. There are no planned interim analyses. This is a comparative effectiveness trial of two acceptable models of care.

#### Plans for communicating important protocol amendments to relevant parties (e.g., trial participants, ethical committees) {25}

Any protocol amendments will be discussed with our study sponsor (PCORI) and the UC Davis Health human subjects review committee. Relevant changes to the protocol will be shared with all staff responsible for carrying out study operations, including clinical staff, transfer center staff, the research analyst team, and clinical research coordinators.

#### Dissemination plans {31a}

At the end of the study, the principal investigator will oversee the development and dissemination of an electronic newsletter with all study findings in language appropriate for the general public. We will also invite study participants to attend a 1-day community lunch (either by video or in-person), hosted at UC Davis and organized by the UC Davis CHPR, to meet all study investigators and to discuss findings from the research study. The CHPR has conducted similar events for study participants and have found that these events are well attended and foster productive dialogue between researchers and community participants resulting in increased knowledge and stronger relationships. The results of the research will also be disseminated at international conferences and through peer-reviewed research publications.

## Discussion

Following the conclusion of this trial, our results will better inform level I pediatric trauma centers and non-pediatric trauma centers across the country and globe on how to improve their systems of care for injured children, specifically with regard to the use of telemedicine. The results will help inform how communication and shared decision-making between parents/families and clinical staff from both the transferring and receiving hospitals impact measures of parental/guardian experiences of care and distress following childhood injury. Our results will also better inform how using the telephone (the current standard of pediatric trauma care) versus using telemedicine (the VPTC model of care) might impact 30-day healthcare utilization, parent/family out-of-pocket costs, transfer rates, and parent/family distress following childhood injury.

## Trial status

Protocol version 5, 2021-05-27

The recruitment for this study began on November 30, 2020. Recruitment and data collection end on March 14, 2023.

## Data Availability

Any data required to support the protocol can be supplied on request.

## Abbreviations

CHPR: Center for Healthcare Policy and Research
ED: Emergency department
iPCQ: iMTA Productivity Cost Questionnaire
PCORI: Patient-Centered Outcomes Research Institute
PECARN: Pediatric Emergency Care Applied Research Network
PRIME: Pediatric Research in Injuries and Medical Emergencies
VPTC: Virtual Pediatric Trauma Center
